# Sequential Production of ᴅ-xylonate and Ethanol from Non-Detoxified Corncob at Low-pH by *Pichia kudriavzevii* via a Two-Stage Fermentation Strategy

**DOI:** 10.3390/jof7121038

**Published:** 2021-12-03

**Authors:** Hao Ji, Ke Xu, Xiameng Dong, Da Sun, Libo Jin

**Affiliations:** 1 Institute of Life Sciences & Engineering Laboratory of Zhejiang Province for Pharmaceutical Development of Growth Factors, Wenzhou University, Wenzhou 325035, China; 20160226@wzu.edu.cn (K.X.); sunday@wzu.edu.cn (D.S.); 20160121@wzu.edu.cn (L.J.); 2 Department of Agriculture and Biotechnology, Wenzhou Vocational College of Science and Technology, Wenzhou 325006, China; dongxiameng@wzvcst.edu.cn

**Keywords:** ethanol, ᴅ-xylonate, *Pichia kudriavzevii*, sequential production, non-detoxified corncob

## Abstract

Improving the comprehensive utilization of sugars in lignocellulosic biomass is a major challenge for enhancing the economic viability of lignocellulose biorefinement. A robust yeast *Pichia kudriavzevii* N-X showed excellent performance in ethanol production under high temperature and low pH conditions and was engineered for ᴅ-xylonate production without xylitol generation. The recombinant strain *P. kudriavzevii* N-X/S1 was employed for sequential production of ᴅ-xylonate and ethanol from ᴅ-xylose, feeding on ᴅ-glucose without pH control in a two-stage strategy of aerobic and shifting micro-aerobic fermentation. Acid-pretreated corncob without detoxification and filtration was used for ᴅ-xylonate production, then simultaneous saccharification and ethanol fermentation was performed with cellulase added at pH 4.0 and at 40 °C. By this strategy, 33.5 g/L ᴅ-xylonate and 20.8 g/L ethanol were produced at yields of 1.10 g/g ᴅ-xylose and 84.3% of theoretical value, respectively. We propose a promising approach for the sequential production of ᴅ-xylonate and ethanol from non-detoxified corncob using a single microorganism.

## 1. Introduction

Lignocellulosic biomass is a promising feedstock for the production of second-generation ethanol and biochemicals owing to its ready availability, economic efficiency and sustainability. ᴅ-glucose, abundantly present in lignocellulosic biomass, can be efficiently converted to ethanol using traditional *Saccharomyces cerevisiae*. ᴅ-xylose, the second most abundant sugar in lignocellulosic biomass, cannot be naturally utilized by *S. cerevisiae*. To improve the economic viability of the lignocellulosic ethanol industry, numerous genetic modifications have been performed to improve the xylose fermentation of *S. cerevisiae* in recent years [1]. In addition to converting ᴅ-xylose into ethanol, an alternative way to improve the lignocellulose-based bioeconomy is to convert ᴅ-xylose into other value-added chemicals [2].

Besides being reduced to xylitol, ᴅ-xylose can also be oxidized to ᴅ-xylonate, an important platform compound that is ranked in the top 30 value-added chemicals published by the U.S. Department of Energy [3]. *S. cerevisiae* was genetically modified to produce ᴅ-xylonate by expressing heterogeneous ᴅ-xylose dehydrogenase encoding genes [4,5]. However, the production rates were much lower than those observed with some bacteria [6], and the engineered *S. cerevisiae* showed poor performance on ᴅ-xylonate production at a low pH. An unconventional yeast *Pichia kudriavzevii* was considered to be an excellent production organism for ᴅ-xylonate, with advantages over *S. cerevisiae* due to its remarkable tolerance towards ᴅ-xylonate, low pH stress and lignocellulosic inhibitors [7,8]. Moreover, *P. kudriavzevii* also showed a higher capacity for ethanol production from non-detoxified lignocelluosic biomass than *S. cerevisiae* [9,10], making it a potential candidate host for improving the lignocellulosic-based bioeconomy.

Due to the competitive inhibition of ᴅ-xylose transport by ᴅ-glucose, it is difficult to produce ethanol and xylose derivatives simultaneously. Some integrated strategies are proposed to separately utilize ᴅ-xylose and ᴅ-glucose by different microorganisms. In previous studies, *Gluconobacter oxydans* and *S. cerevisiae* were employed to produce ᴅ-xylonate using xylose-rich hydrolysates from the pretreated biomass, and ethanol using enzymatic hydrolyzates from glucan residue, respectively [11,12]. Instead of two separate processes, a combination of these microorganisms enables the synthesis of two products in the same bioreactor, saving the time and energy consumption required for sterilization of the bioreactor and growth media, reducing time for the preparation of equipment, etc. Some efforts have been made regarding the sequential production of xylitol and ethanol from non-detoxified acid-pretreated corncob using single robust yeast strains [13,14]. In these studies, two-stage fermentation comprising aerobic xylitol production and simultaneous saccharification and micro-anaerobic ethanol fermentation were performed directly using acid-pretreated corncob without filtration, making the lignocellulosic ethanol industry more economical.

In this study, we constructed an engineering *P. kudriavzevii* strain for ᴅ-xylonate production without xylitol by replacing the ᴅ-xylose reductase gene with a ᴅ-xylose dehydrogenase gene using the *URA3* pop-out system. The recombinant strain *P. kudriavzevii* N-X/S1 exhibited excellent ᴅ-xylonate and ethanol productivity at low pH conditions using ᴅ-xylose and ᴅ-glucose medium, respectively. A two-stage fermentation strategy was then proposed for sequential production of ᴅ-xylonate and ethanol from non-detoxified acid-pretreated corncob at low pH. Our work offers an alternative approach to improve the economic viability of lignocellulose biorefinement.

## 2. Materials and Methods

### 2.1. Strains and Medium

*P. kudriavzevii* N-X (CCTCC M2017759) was isolated from Kazakh cheese and *S. cerevisiae* W13 was isolated from grape skin, and they were deposited in our lab at −80 °C. The yeast strains were cultured in YPD medium (20 g/L ᴅ-glucose, 20 g/L peptone, 10 g/L yeast extract) for maintenance. Minimal medium (MM, 6.7 g/L yeast nitrogen base without amino acids, 20 g/L ᴅ-glucose) was used for selecting yeast transformants. Minimal medium supplemented with 0.1 g/L uracil and 1 g/L 5-Fluoroorotic acid (5-FOA) was used for the selection of uracil auxotrophic mutant. *Escherichia coli* DH5α was cultured in Luria–Bertani (LB) medium (10 g/L peptone, 5 g/L yeast extract, 10 g/L NaCl) for plasmid construction and propagation.

### 2.2. Strain Construction

For efficient genetic manipulation in *P. kudriavzevii* N-X, we constructed a uracil auxotrophic mutant using a previously reported method [15]. To construct a ᴅ-xylonate producing strain, the ᴅ-xylose reductase gene *XYL1* was replaced with the codon-optimized ᴅ-xylose dehydrogenase gene *xylB* (Table S1) from *Caulobacter crescentus* using the *URA3* pop-out system (Figure S1). In this case, the *xylB* gene was synthesized by Genewiz (Suzhou, China), and cloned under the *TDH* promoter. The *URA3* pop-out system was then constructed in the plasmid pMD19T (Takara Bio, Dalian, China) using a ClonExpress^®^ MultiS One Step Cloning Kit (Vazyme, Nanjing, China). The primers used in this study were listed in Table S2. The obtained plasmid was lined and introduced into the uracil auxotrophic mutant by the previous reported method [14]. The recombinant strain *P. kudriavzevii* N-X/S1 was obtained from the MM medium.

### 2.3. Ethanol and ᴅ-xylonate Fermentation in Flasks

To evaluate the ethanol fermentation capacity under various environmental stresses, *P. kudriavzevii* N-X was cultured in 250 mL flasks containing 100 mL YP medium supplemented with different concentrations of ᴅ-glucose. The initial pH of the culture medium was adjusted to 2.0–6.0 using HCl before autoclaving, the temperature was set at 30–45 °C, and the rotation speed of the shaker was set at 100 rpm. *S. cerevisiae* W13 was used as a control for ethanol fermentation at different initial pH conditions and 30 °C. 

For ᴅ-xylonate production, the recombinant *P. kudriavzevii* N-X/S1 was cultured in 250 mL flasks containing 50 mL YPD medium supplemented with 50 g/L ᴅ-xylose at 37 °C. The rotation speed of the shaker was set at 250 rpm, and the initial pH of the medium was adjusted to 3.0 and 5.5, respectively. All the experiments were repeated in triplicate.

### 2.4. Materials and Acid Pretreatment

Corncobs used in this study were collected from a farm in Zhejiang province, China. After air drying to constant mass, the material was ground by a hammer mill and then filtered through a 10-mesh screen. The composition of raw material was determined as 30.8 ± 0.51% xylan, 42.5 ± 0.22% glucan and 14.5 ± 0.35% lignin by the protocol from National Renewable Energy Laboratory (NERL) [16]. The cellulase CE1000 was obtained from Jiangsu Boli Bioproducts Co., Ltd. in China, and its activity (92.5 FPU/g) was determined following the procedure recommended by the NREL [17].

The acid pretreatment was performed in a stainless-steel reactor following the method described in a previous study [13]. Briefly, milled corncob was slurred at a solid–liquid ratio of 1:3 with 0.5% (*w*/*w*) sulphuric acid and 1.5% (*w*/*w*) phosphoric acid, and then autoclaved at 125 °C for 1 h. Since a high solid–liquid ratio might cause insufficient mass transfer [18], sterile water was added to the mixture to adjust the solid–liquid ratio of 1:8, after acid pretreatment. The pH of corncob slurry was adjusted to 4.0. To evaluate the combined effects of inhibitors on *P. kudriavzevii* N-X/S1 under pH 4.0, the medium was prepared using the liquid fraction of lignocellulosic slurry supplemented with 10 g/L yeast extract, 20 g/L peptone and ᴅ-glucose to 20 g/L, and then filtrated through a 0.22 μm membrane for sterilization.

### 2.5. Two-Stage Fermentations

The recombinant *P. kudriavzevii* N-X/S1 was cultured in a 500 mL flask containing 100 mL YPD medium for 16 h at 37 °C and 250 rpm for seed preparation. For two-stage fermentation with pure sugars, the seed culture was inoculated at 5% (*v*/*v*) into the 2 L YPD medium supplemented with 50 g/L ᴅ-xylose at an initial pH of 5.5 in a 5 L bioreactor (BIOTECH-5BG, Bxbio, Shanghai, China). At the first stage, aerobic fermentation was performed at 40 °C with an agitation speed of 500 rpm and an aeration rate of 1.5 vvm. After ᴅ-xylose was depleted, a glucose stock of 500 g/L was fed into the culture at one time until the final concentration reached 150 g/L, then a shift to micro-anaerobic conditions was performed by controlling the aeration rate at 0.2 vvm for ethanol production. The pH value was not controlled during the whole fermentation process.

For two-stage fermentation using non-detoxified corncob, 10 g/L yeast extract and 20 g/L peptone was added to the acid-pretreated corncob slurry, and ᴅ-glucose was added to 20 g/L for cell growth at the first stage. After all of the ᴅ-xylose was converted to ᴅ-xylonate at 48 h, 20 FPU/g corncob was added and simultaneous saccharification and fermentation was performed in micro-anaerobic conditions (aeration rate at 0.2 vvm and 40 °C). The pH was controlled at 4.0 using 5 M NaOH.

### 2.6. Analytical Methods

For the determination of dried cell weight (DCW), 2 mL samples were collected in a pre-dried centrifuge tube, washed twice and then dried in a vacuum drying oven at 105 °C. Sugars, organic acids, furfural and 5-HMF were identified and quantified using a high-performance liquid chromatograph (HPLC; Hitachi, Tokyo, Japan) equipped with refractive index (RI) and UV detectors and an Aminex HPX-87H column (Bio-Rad, Hercules, CA, USA), following the method described in our precious study [8].

## 3. Results

### 3.1. Ethanol Fermentation Capacity of P. kudriavzevii N-X

*P. kudriavzevii* strains exhibited greater ethanol production capacity than conventional *S. cerevisiae* under some harsh conditions, such as high temperature, hyperosmotic stress and inhibitor stress [10,19,20]. The strain *P. kudriavzevii* N-X reported in our previous study showed excellent low-pH stress tolerance, but its ethanol fermentation capacity has not been evaluated yet [21]. Herein, we firstly evaluated the capacity of *P. kudriavzevii* N-X for ethanol production under various conditions in flasks. *P. kudriavzevii* was reported to have high osmotolerance, tolerating up to 48% (*w*/*v*) ᴅ-glucose, but the ethanol productivity is usually affected under high ᴅ-glucose concentrations [9,22,23]. In this study, *P. kudriavzevii* N-X showed a similar yield and productivity of ethanol in the presence of 100 and 150 g/L ᴅ-glucose, but the ethanol fermentation capacities were significantly reduced when the initial ᴅ-glucose concentration was further increased to 200 g/L (Table S3). *P. kudriavzevii* N-X can ferment at high temperatures, but its ethanol production capacity at 45 °C was not superior to that of some reported *P. kudriavzevii* strains [19,23]. The maximum ethanol yield (94.3% of theoretical yield) and productivity (3.01 g/L/h) were obtained at 40 °C (Table S4).

To explore the possibility of ethanol fermentation in low pH conditions, batch cultivations were performed under various initial pH conditions. The results showed that *P. kudriavzevii* N-X maintained high ethanol production capacity in the initial pH range of 3.0–6.0; it produced 67.1 g/L ethanol at 87.7% of the theoretical yield and its productivity was 2.40 g/(L·h) at pH 3.0, while *S. cerevisiae* W13 produced only 44.6 g/L ethanol at 58.3% of the theoretical yield and its productivity was 1.24 g/(L·h) (Table 1). Moreover, *P. kudriavzevii* N-X performed much better than *S. cerevisiae* W13 at lower pH levels, the yield of ethanol was decreased to 49.2% of the theoretical yield at pH 2.0, while *S. cerevisiae* W13 did not grow at all. The decrease in the ethanol fermentation capacity of *P. kudriavzevii* N-X under extreme acidic conditions may be due to the decreased count of viable cells and prolonged lag phase (data not shown). Additionally, *P. kudriavzevii* N-X grows as large aggregates at pH levels below 2.5, which affects nutrient transfer and oxygen diffusion, and so is not suited to low-pH fermentation [21].

### 3.2. Construction of a ᴅ-xylonate-Producing P. kudriavzevii

**Figure 1:**
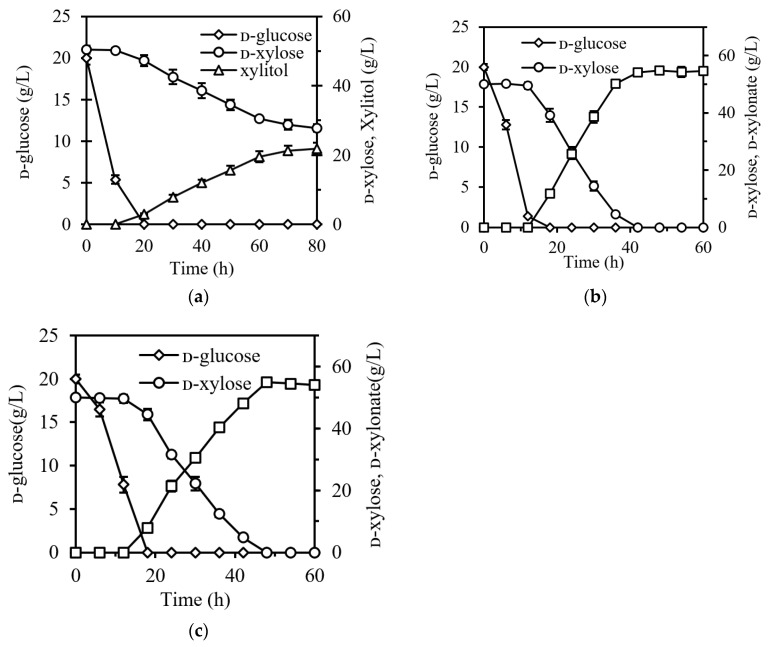
Time course of xylitol and ᴅ-xylonate production in the YPD medium containing 50 g/L ᴅ-xylose. (**a**) Xylitol production of *P. kudriavzevii* N-X. ᴅ-xylonate production of *P. kudriavzevii* N-X/S1 at initial pH 5.5 (**b**) and pH 3.0 (**c**), respectively. Error bars represent ± SD.

*P. kudriavzevii* is an excellent host for ᴅ-xylonate production utilizing ᴅ-xylose because it has a high tolerance for and does not catabolize ᴅ-xylonate [7,8]. However, some *P. kudriavzevii* strains could also metabolize ᴅ-xylose for cell growth or convert xylose into xylitol, which might reduce the yield of ᴅ-xylonate from ᴅ-xylose. It was found that *P. kudriavzevii* strains exhibited different abilities to utilize xylose, although their genomes contain the three key enzymes required for ᴅ-xylose utilization [24]. Early taxonomic studies claimed that *P. kudriavzevii* could not assimilate and ferment ᴅ-xylose, but some strains were reported to be able to metabolize ᴅ-xylose for growth [7,22,25]. In a previous study, the yield of ᴅ-xylonate from recombinant *P. kudriavzevii* VTT-C-12903 reached up to 0.9–1.0 g/g ᴅ-xylose, but a certain proportion of ᴅ-xylose was still consumed for cellular growth and xylitol production [7]. In this study, we evaluated the ᴅ-xylose metabolic capacity of *P. kudriavzevii* N-X before constructing the ᴅ-xylonate producing strain. It showed that *P. kudriavzevii* N-X could not grow in the medium using ᴅ-xylose as a sole carbon source, but it converted ᴅ-xylose to xylitol at a yield of 0.4 g/g in the presence of ᴅ-glucose as carbon source (Figure 1a). These results indicate that the xylose catabolic pathway of *P. kudriavzevii* N-X is nonfunctional, but the gene encoding ᴅ-xylose reductase is at least active. Hence, to produce ᴅ-xylonate and at the same time eliminate the production of xylitol, we replaced the two copies of ᴅ-xylose reductase gene *XYL1* in the uracil auxotrophic mutant of *P. kudriavzevii* N-X with the codon-optimized ᴅ-xylose dehydrogenase gene from *Caulobacter crescentus* using the *URA3* pop-out system. A recombinant strain, *P. kudriavzevii* N-X/S1, was obtained from the selective medium and confirmed by PCR (data not shown).

Batch fermentation was then performed under aerobic conditions in flasks containing YPD medium supplemented with 50 g/L ᴅ-xylose. As shown in Figure 1b, the recombinant strain *P. kudriavzevii* N-X/S1 produced 54.8 g/L ᴅ-xylonate within 40 h at a yield of 1.1 g/g and a rate of 1.37 g L^−1^ h^−1^. The yield was higher than that of VTT-C-12903 under the same conditions, probably due to the absence of native xylose catabolism and the blocking of the conversion of ᴅ-xylose to xylitol. Moreover, the recombinant strain converted all the ᴅ-xylose to ᴅ-xylonate at a rate of 1.13 g L^−1^ h^−1^ at pH 3.0 (Figure 1c), showing good potential in low-pH ᴅ-xylonate production.

### 3.3. Sequential Production of ᴅ-xylonate and Ethanol without pH Control by a Two-Stage Strategy

**Figure 2:**
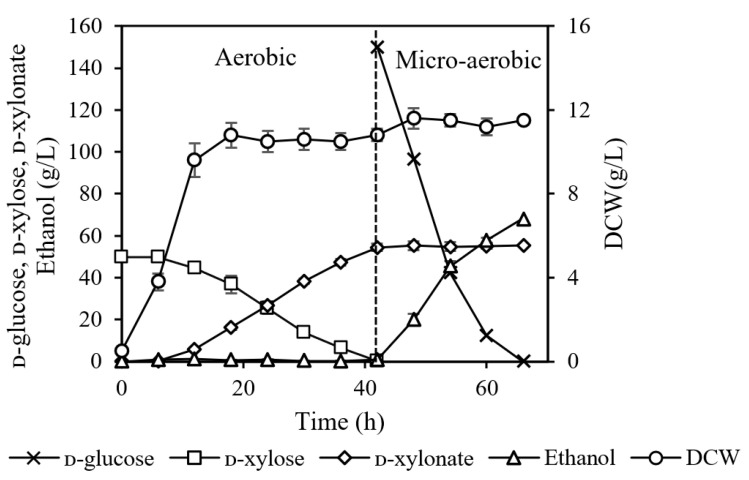
Sequential production of ᴅ-xylonate and ethanol from pure ᴅ-xylose and ᴅ-glucose medium by a two-stage fermentation.

The above results showed that recombinant *P. kudriavzevii* can efficiently produce ethanol and ᴅ-xylonate at low pH conditions, demonstrating its great potential for biotechnological conversion using lignocellulosic biomass rich in ᴅ-glucose and ᴅ-xylose. However, simultaneous fermentation of ᴅ-glucose and ᴅ-xylose cannot be realized because the presence of ᴅ-glucose strongly inhibits ᴅ-xylose transport [26]. It was reported that the two-stage fermentation strategy removed the glucose effect and enabled sequential consumption of ᴅ-xylose and ᴅ-glucose for the production of ethanol or co-production of ethanol and xylitol [13,27,28]. Herein, we investigated the continuous production of ᴅ-xylonate and ethanol by *P. kudriavzevii* N-X/S1 using pure ᴅ-xylose and ᴅ-glucose via a two-stage and fed-batch fermentation. In the first stage, *P. kudriavzevii* N-X/S1 was cultured in YPD medium containing 50 g/L ᴅ-xylose under aerobic conditions, resulting in high biomass yield and an accumulation of ᴅ-xylonate. After ᴅ-xylose depletion, a shift to micro-anaerobic ethanol fermentation was performed, with 150 g/L ᴅ-glucose being fed into the culture. As shown in Figure 2, 55.3 g/L ᴅ-xylonate was produced with a yield of 1.10 g/g ᴅ-xylose in 42 h aerobic cultivation. The pH value of broth was 3.10 at the end of the first stage and it was not adjusted during the subsequent ethanol fermentation, since *P. kudriavzevii* N-X/S1 maintained a high ethanol fermentation capacity at a pH value around 3.0. In the second stage, ethanol was produced during 42–60 h, and the yield and productivity reached 89.2% of theoretical yield and a rate of 2.84 g/(L·h), respectively. Importantly, the ᴅ-xylonate accumulating in the broth was not utilized any more during the whole fermentation process, as reported in a previous study [7]. In this case, by regulating the aerobic and micro-anaerobic conditions, ᴅ-xylose and ᴅ-glucose consumption was separated into two stages, avoiding the competitive inhibition of sugar transport. Fermentation without pH control reduces the cost of the base needed for acid neutralization, minimizes the risk of contamination and simplifies downstream engineering [29]. Moreover, ᴅ-xylonate and ethanol can be easily separated by distillation in the downstream processing because of their different boiling points. These results indicated that the sequential production of ᴅ-xylonate and ethanol in a single bioreactor can be achieved via a two-stage strategy.

### 3.4. Sequential Production of ᴅ-xylonate and Ethanol Using Non-Detoxified Corncob

**Figure 3:**
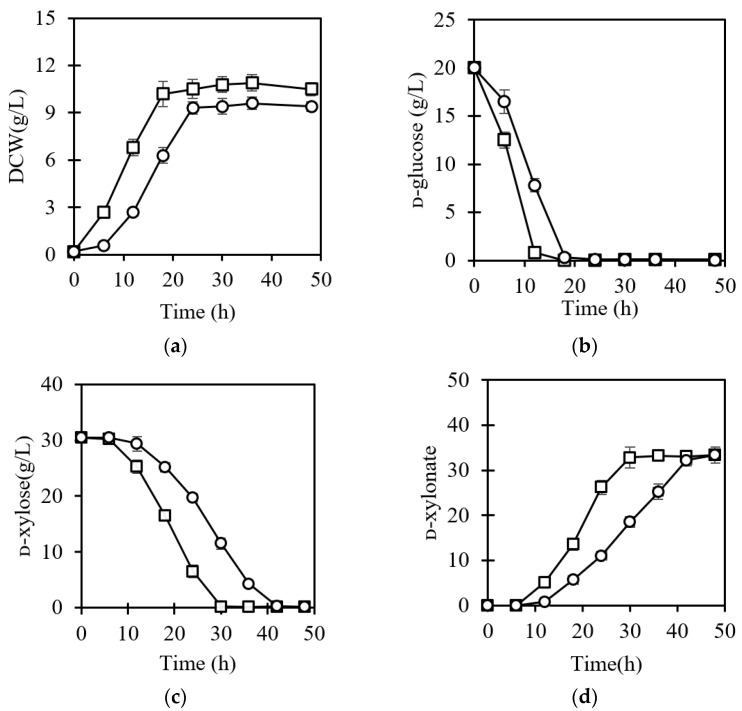
Effect of combined inhibitors on cell growth (**a**), ᴅ-glucose (**b**) and ᴅ-xylose consumption (**c**) and ᴅ-xylonate (**d**) production of *P. kudriavzevii* N-X/S1 at pH 4.0. The cells were cultured in the medium prepared by the liquid fraction of acid-pretreated corncob without detoxification (open circles) or YPD medium supplemented with 30.4 g/L ᴅ-xylose (open squares).

*P. kudriavzevii* strains exhibited multi-tolerance to inhibitors and have been used in ethanol production from non-detoxified lignocellulosic biomass [10,19,22,30]. Nevertheless, ᴅ-xylose in the lignocellulosic hydrolysates is usually retained because it cannot be efficiently consumed by *P. kudriavzevii*, resulting in reduced economic feasibility. Herein, aerobic ᴅ-xylonate and micro-anaerobic ethanol fermentation from non-detoxified corncob were integrated using *P. kudriavzevii* N-X/S1 via a two-stage strategy. In this case, the liquid fraction of acid-pretreated corncob without detoxification contained 30.4 g/L ᴅ-xylose, and 2.80 g/L ᴅ-glucose, 0.55 g/L furfural, 0.38 g/L 5-HMF and 1.20 g/L acetic acid. For low-pH fermentation, the pH value of the culture would be controlled at 4.0, since the commercial acidic cellulases usually have low activity at a lower pH [31]. Previous studies suggested that *P. kudriavzevii* strains were tolerant of the inhibitors at concentrations much higher than those in our acid-pretreated corncob [19,30]. To evaluate the combined effects of inhibitors and low pH on *P. kudriavzevii* N-X/S1, we compared the cell growth and ᴅ-xylonate production in flasks using the liquid fraction of non-detoxified acid-pretreated corncob hydrolysate and pure ᴅ-xylose medium. ᴅ-glucose was supplemented with an additional 20 g/L because the amount in the acid-pretreated hydrolysate was not enough to support cell growth. As shown in Figure 3, lower cell growth and a prolonged lag phase were observed with non-detoxified acid-pretreated hydrolysate as substrate. The accumulation of ᴅ-xylonate was slower than when using pure ᴅ-xylose as substrate, but the yield was maintained at 1.10 g/g. These results suggested that the combined inhibitors in corncob hydrolysate had limited the repression effect on *P. kudriavzevii* N-X/S1 at pH 4.0.

**Figure 4:**
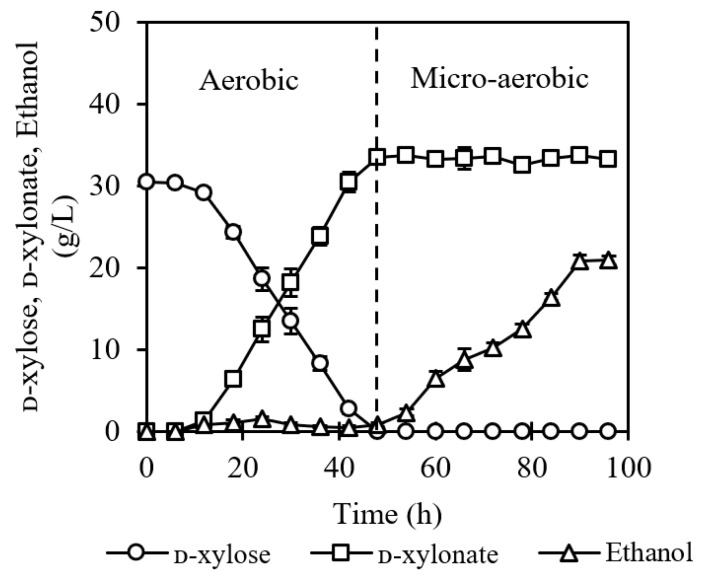
Sequential production of ᴅ-xylonate and ethanol from non-detoxified acid-pretreated corncob by a two-stage fermentation strategy.

The two-stage fermentation was carried out at 40 °C, which is the optimum temperature for ethanol fermentation of *P. kudriavzevii* N-X/S1, and the same temperature was maintained for saccharification. ᴅ-glucose concentration was not shown because it was consumed immediately during the saccharification process. As shown in Figure 4, ᴅ-xylose present in the acid-pretreated corncob slurry was totally converted to 33.5 g/L ᴅ-xylonate at pH 4.0 after 48 h aerobic incubation. In the case of solids loading, the depletion of ᴅ-xylose was delayed, probably due to insufficient mass transfer. After acidic cellulase application, about 80.2% of glucan was hydrolyzed to monomeric ᴅ-glucose and 20.8 g/L ethanol was produced by simultaneous saccharification and micro-anaerobic fermentation within another 48 h, at 84.3% of the theoretical yield. The mass balance of the two-stage fermentation by corncob showed that 26.8 g ᴅ-xylonate and 16.6 g ethanol were produced from 100 g corncob. This is the first study reporting the sequential production of ᴅ-xylonate and ethanol from non-detoxified lignocellulosic biomass at a low pH using a single microorganism. In previous studies, *S. cerevisiae* and *G. oxydans* were employed for aerobic ᴅ-xylonate and micro-anaerobic ethanol production from lignocellulosic biomass by two independent operations [11,12]. By contrast, the sequential production of ᴅ-xylonate and ethanol using *P. kudriavzevii* in the same container simplifies manufacturing, minimizes contamination and reduces wastewater generation. Limited by the activity of commercial cellulase, this two-stage fermentation is not feasible for operating under lower pH conditions. Nonetheless, the cost of pH titration is still effectively controlled in this study.

## 4. Conclusions

In this study, a recombinant strain, *P. kudriavzevii* N-X/S1, was constructed to produce ᴅ-xylonate at a high yield and productivity without xylitol production. This strain also showed high tolerance of elevated temperatures, low pH conditions and lignocellulosic inhibitor stresses, and demonstrated high efficiency of ethanol productivity at elevated temperatures and in low pH conditions. Aerobic ᴅ-xylonate production and micro-anaerobic ethanol fermentation can be integrated in the same bioreactor using this single microorganism via a two-stage strategy. Furthermore, ᴅ-xylonate and ethanol were sequentially produced from whole slurry non-detoxified corncob at pH 4.0—a result which shows great potential for industrial applications.

## Figures and Tables

**Table 1:** Ethanol production of *Pichia kudriavzevii* N-X and *Saccharomyces cerevisiae* W13 under different initial pH conditions.

pH	*P. kudriavzevii* N-X				*S. cerevisiae* W13			
	Fermentation Time (h)	Ethanol (g/L)	% of Theoretical Yield	Productivity (g/L/h)	Fermentation Time (h)	Ethanol (g/L)	% of Theoretical Yield	Productivity (g/L/h)
2.0	48	37.6 ± 2.8	49.2 ± 3.6	0.78 ± 0.06	-	-	-	-
2.5	32	52.7 ± 2.0	68.9 ± 2.5	1.65 ± 0.06	48	25.2 ± 1.0	33.0 ± 2.4	0.53 ± 0.02
3.0	28	67.1 ± 1.7	87.7 ± 2.2	2.40 ± 0.06	36	44.6 ± 1.9	58.3 ± 2.4	1.24 ± 0.06
4.0	24	70.5 ± 2.0	92.1 ± 2.6	2.94 ± 0.08	28	66.3 ± 0.9	86.7 ± 1.2	2.34 ± 0.03
5.0	24	71.4 ± 0.9	93.3 ± 1.1	2.98 ± 0.04	24	67.3 ± 1.1	88.0 ± 1.4	2.80 ± 0.05
6.0	24	68.2 ± 2.7	89.2 ± 3.5	2.84 ± 0.11	24	68.4 ± 1.0	89.4 ± 1.3	2.85 ± 0.04

The experiments were repeated in triplicate. The data present the average ± SD.

## Data Availability

Not applicable.

## Supplementary Materials

The following are available online at https://www.mdpi.com/article/10.3390/jof7121038/s1, Figure S1: Schematic diagram of expression *xylB* at the *XYL1* locus using the URA3 pop-out system, Table S1: Sequence of NAD+ -dependent xylose dehydrogenase gene *xylB*, Table S2: Primers used in this study, Table S3: Ethanol production of *P. kudriavzevii* N-X in YP medium containing different concentrations of ᴅ-glucose by shaking flasks at 100 rpm and 37 °C, Table S4: Ethanol production of *P. kudriavzevii* N-X in YP medium containing 150 g/L ᴅ-glucose glucose by shaking flasks at 100 rpm and different temperatures.
