# Supplementary material for: Sequential Production of ᴅ-xylonate and Ethanol from Non-Detoxified Corncob at Low-pH by Pichia kudriavzevii via a Two-Stage Fermentation Strategy
Source: J Fungi (Basel). 2021 Dec 3;7(12):1038. doi: 10.3390/jof7121038 (PMC8709110; doi:10.3390/jof7121038)

Table S1 Sequence of NAD^{+} -dependent xylose dehydrogenase gene *xylB*

Name	Sequence
Native *xylB*	ATGTCCTCAGCCATCTATCCCAGCCTGAAGGGCAAGCGCGTCGTCATCACCGGC<br>GGCGGCTCGGGCATCGGGGCCGGCCTCACCGCCGGCTTCGCCCGTCAGGGCGC<br>GGAGGTGATCTTCCTCGACATCGCCGACGAGGACTCCAGGGCTCTTGAGGCCG<br>AGCTGGCCGGCTCGCCGATCCCGCCGGTCTACAAGCGCTGCGACCTGATGAAC<br>CTCGAGGCGATCAAGGCGGTCTTCGCCGAGATCGGCGACGTCGACGTGCTGGT<br>CAACAACGCCGGCAATGACGACCGCCACAAGCTGGCCGACGTGACCGGCGCC<br>TATTGGGACGAGCGGATCAACGTCAACCTGCGCCACATGCTGTTCTGCACCCA<br>GGCCGTCGCGCCGGGCATGAAGAAGCGTGGCGGCGGGGCGGTGATCAACTTC<br>GGTTCGATCAGCTGGCACCTGGGGCTTGAGGACCTCGTCCTCTACGAAACCGC<br>CAAGGCCGGCATCGAAGGCATGACCCGCGCGCTGGCCCGGGAGCTGGGTCCC<br>GACGACATCCGCGTCACCTGCGTGGTGCCGGGCAACGTCAAGACCAAGCGCC<br>AGGAGAAGTGGTACACGCCCGAAGGCGAGGCCCAGATCGTGGCGGCCCAATG<br>CCTGAAGGGCCGCATCGTCCCGGAGAACGTCGCCGCGCTGGTGCTGTTCCTGG<br>CCTCGGATGACGCGTCGCTCTGCACCGGCCACGAATACTGGATCGACGCCGGC<br>TGGCGTTGA
Codon optimized *xylB*	ATGTCCTCCGCAATCTACCCATCCTTGAAGGGTAAGCGTGTCGTTATCACTGGA<br>GGTGGTTCTGGTATCGGTGCTGGTTTGACAGCTGGTTTCGCTCGTCAAGGTGCT<br>GAGGTCATCTTCTTGGACATTGCTGACGAGGATTCCCGTGCTTTGGAGGCTGAA<br>TTAGCTGGTTCCCCTATCCCACCAGTCTACAAGCGTTGCGACTTGATGAACTTG<br>GAGGCAATCAAGGCAGTCTTCGCAGAGATCGGAGATGTCGACGTCTTGGTCAA<br>CAACGCTGGTAACGACGACAGACACAAGTTGGCTGACGTCACTGGTGCTTACT<br>GGGACGAGCGTATTAACGTTAACTTGAGACACATGTTGTTCTGCACTCAAGCA<br>GTTGCACCCGGTATGAAGAAGAGAGGTGGTGGTGCTGTCATCAACTTCGGTTC<br>CATCTCTTGGCACTTGGGATTGGAGGACTTGGTCTTGTACGAGACTGCAAAGG<br>CTGGTATCGAGGGTATGACTAGAGCATTGGCTCGTGAGTTGGGTCCAGACGAC<br>ATCCGTGTCACTTGCGTTGTCCCCGGTAACGTTAAGACTAAGCGTCAAGAAAA<br>GTGGTACACACCAGAAGGAGAGGCACAGATTGTCGCTGCTCAGTGCTTGAAG<br>GGTCGTATTGTCCCAGAGAACGTCGCAGCTTTGGTCTTGTTCTTGGCATCCGAC<br>GACGCTTCCTTGTGTACTGGTCACGAGTATTGGATCGATGCTGGTTGGCGTTAA

Table S2 Primers used in this study.

Name	Primer sequences	Products
XYL1&p-F	CACCATTCCCACTATTATCACC	*XYL1* promoter and *XYL1* ORF
XYL1&p-R	CGACTCTAAGGTTGAAAACACATT	
TDHpro-F	tattttaaagcttaaacacaGGGCTAGATTTCGATATGGATATGG	*TDH* promoter
TDHpro-R	ggtagattgcggaggacatTTTTTGTAATTGTGTTTGTTTGTGTGT	
xylB-F	aATGTCCTCCGCAATCTACCCA	*xyl*B
xylB-R	caccatTTAACGCCAACCAGCATCG	
XYL1ter-F	ctggttggcgttaaATGGTGTTAGTCTGATCTAATGCAACA	*XYL1* terminator
XYL1ter-R	atgtcaaccttccctgtTGCCGTTGCCAGAGCTATTG	
URA3-F	gcaACAGGGAAGGTTGACATTGTCTAGC	*URA3* expression cassettes
URA3-R	ggatggtccaattcaaggaaAACACTTAGAATACGCGGAACAATC	
TXYL-F	TTCCTTGAATTGGACCATCCA	*XYL1* 3’flank&T-vector&*XYL1* promoter
TXYL-R	TGTGTTTAAGCTTTAAAATAGTTTAGTTTGG	

Table S3 Ethanol production of *P. kudriavzevii* N-X in YP medium containing different concentrations of D-glucose by shaking flasks at 100 rpm and 37 °C.

D-glucose concentrations	Fermentation time (h)	Ethanol (g/L)	% of theoretical yield	Productivity(g/L/h)
100	16	46.8±0.51	91.8±1.0	2.93±0.03
150	24	70.5±1.95	92.1±2.6	2.94±0.08
200	36	78.6±1.50	77.1±1.5	2.46±0.05

Table S4 Ethanol production of *P. kudriavzevii* N-X in YP medium containing 150 g/L D-glucose glucose by shaking flasks at 100 rpm and different temperatures.

Temperature (°C)	Fermentation time (h)	Ethanol (g/L)	% of theoretical yield	Productivity(g/L/h)
30	28	60.1±1.21	78.6±1.6	2.15±0.04
35	24	66.4±1.43	86.8±1.9	2.77±0.06
40	24	72.2±1.35	94.3±1.7	3.01±0.06
42	28	63.2±2.40	82.6±3.1	2.26±0.08
45	36	19.2±3.42	25.1±4.5	0.53±0.08

Figure S1 Schematic diagram of expression *xyl* B at *XYL1* locus using the *URA3* pop-out system.

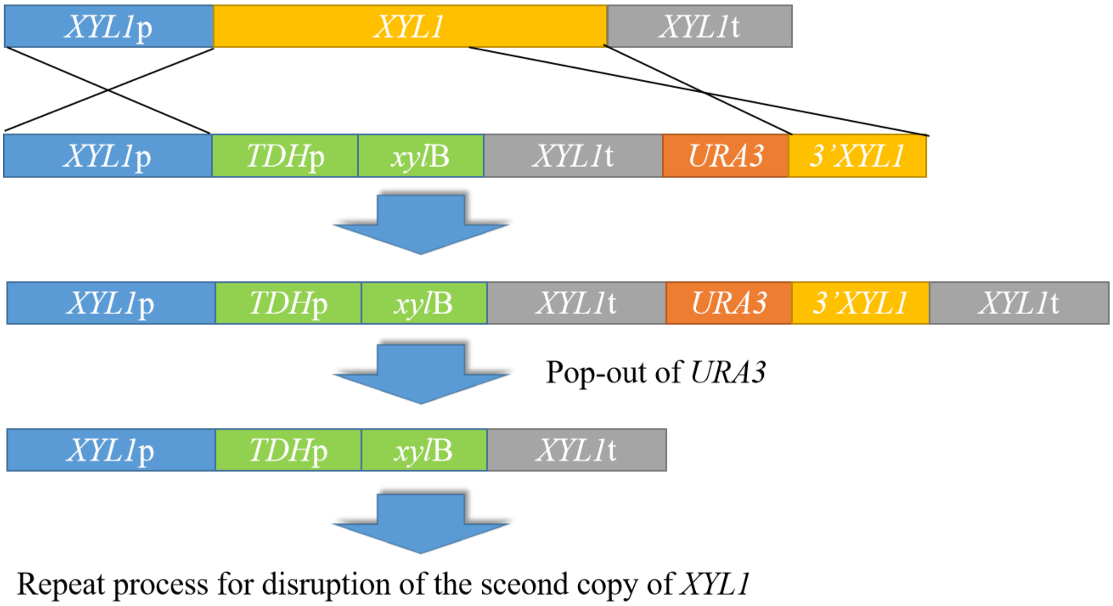

Supplement: Supplementary file 1 [file jof-07-01038-s001.zip › jof-1485752-SI.pdf]
